# *Platycodon grandiflorum* Saponins Ameliorate Cisplatin-Induced Acute Nephrotoxicity through the NF-κB-Mediated Inflammation and PI3K/Akt/Apoptosis Signaling Pathways

**DOI:** 10.3390/nu10091328

**Published:** 2018-09-19

**Authors:** Weizhe Zhang, Jingang Hou, Xiaotong Yan, Jing Leng, Rongyan Li, Jing Zhang, Jingjing Xing, Chen Chen, Zi Wang, Wei Li

**Affiliations:** 1 Department of Chinese Materia Medica, College of Chinese Medicinal Materials, Jilin Agricultural University, Changchun 130118, China; WeizheZhang1215@163.com (W.Z.); houjg2014@kaist.ac.kr (J.H.); yanxiaotong0707@126.com (X.Y.); lj170907@126.com (J.L.); lyr19930612@126.com (R.L.); zhjing0701@163.com (J.Z.); m13944152775_3@163.com (J.X.); wangzi8020@126.com (Z.W.); 2 Intelligent Synthetic Biology Center, Daejeon 34141, Korea; 3 School of Biomedical Sciences, University of Queensland, Brisbane 4072, Australia; chen.chen@uq.edu.au

**Keywords:** *Platycodon grandiflorum* saponins, cisplatin, nephrotoxicity, NF-κB, PI3K/Akt/Apoptosis, inflammation

## Abstract

Although cisplatin is a potent chemotherapeutic agent against cancers, its clinical application is seriously limited by its severe side effects of nephrotoxicity. Previous studies reported that saponins isolated from the roots of *Platycodon grandiflorum* (PGS) exerted protective effects in various animal models of renal injury, with no confirmation on cisplatin-induced injury. This study was designed to investigate the protective effect of PGS (15 and 30 mg/kg) on cisplatin-induced kidney injury in mice. The levels of serum creatinine (CRE) and blood urea nitrogen (BUN), and renal histopathology demonstrated the protective effect of PGS against cisplatin-induced kidney injury. PGS exerted anti-inflammation effects via suppressing nuclear factor-kappa B (NF-κB) activation and alleviating the cisplatin-induced increase in inducible nitric oxide synthase (iNOS), cyclooxygenase-2 (COX-2), tumor necrosis factor-α (TNF-α), and interleukin-1β (IL-1β) in kidney tissues. The expressions of phosphorylation of phosphatidylinositol 3-kinase/protein kinase B and its downstream apoptotic factors, such as Bcl-2 and caspase families were regulated by PGS in a dose-dependent manner. In conclusion, PGS exerted kidney protection effects against cisplatin-induced kidney injury by inhibiting the activation of NF-κB and regulating PI3K/Akt/apoptosis signaling pathways in mice.

## 1. Introduction

Cis-diamminedichloroplatinum (Cisplatin) is an efficient platinum-containing chemotherapeutic agent which is widely used to treat various tumors, including testicular, neck, cervical, and non-small cell lung carcinomas [1]. However, its clinic application is limited due to its serious side effects of neurotoxicity, ototoxicity, nausea, and vomiting, and particularly nephrotoxicity and liver dysfunction [2]. The molecular mechanism underling cisplatin-evoked renal injuries is multifactorial [3]. Among them, accumulation of cisplatin in straight proximal and distal curved tubules, along with its interference with nuclear DNA and the mitochondrial respiratory chain are regarded as the major causes [4]. The over-generation of ROS and the decrease in renal antioxidant capacity caused by cisplatin further trigger apoptosis and inflammation in renal tissues [5]. Generally, cisplatin in the renal tubular epithelial cells promotes the expression of inflammatory factors such as TNF-α and IL-1β; meanwhile these factors further generate the inflammatory mediators including iNOS and COX-2 [6]. Recently, research has focused on the development of anti-inflammatory and anti-apoptotic drugs extracted from medicinal plants to manage cisplatin-induced renal injury [7]. Therefore, effective treatment against cisplatin-induced renal injury is searched in cancer therapeutic research.

Phosphatidylinositol 3-kinase (PI3K) is an important intracellular kinase that maintains cell homeostasis. Activated PI3K produces phosphatidylinositol 3,4,5-triphosphate (PIP3) on the plasma membrane to activate Protein kinase B (Akt) and phosphoinositide-dependent kinase (PDK), which further activate Akt protein kinases [8]. Akt (protein kinase B) takes effect in regulating the transduction of biological signals in the cell to regulate cell growth, survival, and differentiation [9,10]. More apoptotic renal tubular epithelial cells were detected in PI3K knockout mice treated by cisplatin, proving the importance of the PI3K/Akt pathway in protecting kidneys [11].

As an early responding factor, NF-κB takes great effect in activating proinflammatory factors [12]. Under inactivation conditions, NF-κB combines with inhibiting NF-κB proteins (IκBs) to form a trimer that is retained in the cytoplasm. Once IκBs are phosphorylated and degraded, NF-κB moves from the cytosol to the nucleus to regulate its target genes [13]. Sahin et al. have proved that NF-κB was activated by cisplatin to trigger the release of proinflammatory cytokines; inhibiting the NF-κB signaling pathway might be a potential target for preventing cisplatin-induced nephrotoxicity [14].

Accumulating evidence has shown that the active compounds from medicinal herbs exert protective effects against nephrotoxicity induced by cisplatin [15]. The root of *Platycodon grandiflorum*, a common medicinal and edible plant, contains many active ingredients, such as steroidal saponins, flavonoids, phenolic acids, and sterols, in which the saponins are regarded as the major active compounds [16]. The root of *P. grandiflorum* (PG) is widely used in traditional Chinese medicine, which exert hepatoprotective and insulin-sensitizing effects [17]. Numerous studies have proven that the saponins of *P. grandiflorum* (PGS) has multiple kinds of excellent pharmacological activities, such as anti-oxidant [18], anti-inflammatory [19], and anti-apoptosis [20]. In our previous studies, Platycodin D (a major saponin in PG) exerted anti-oxidation and anti-inflammation activities in hepatic damage triggered by alcohol [21]. Considering that cisplatin can cause inflammatory response and apoptosis in tubular cells, this study demonstrated the renal-protective effects of PGS cisplatin-induced renal injury via the modulation of NF-κB-mediated inflammation and PI3K/Akt/apoptosis signaling pathways.

## 2. Materials and Methods

### 2.1. Chemical Compounds and Reagents

*Platycodon grandiflorum* (PG) was harvested from the Jilin Agricultural University medicine plantation garden, and identified by Prof. Wei Li. The Voucher specimen was kept in our lab in the College of Chinese Medicinal Materials, Jilin Agricultural University. *Platycodon grandiflorum* saponins (PGS) extracted from the roots of P. grandiflorum were prepared and quantified in our laboratory. Firstly, PGS was extracted three times with 80% ethanol. Followed by, the extracts were separated by AB-8 resin column chromatography. Subsequently, HPLC analysis of PGS was performed by a Hypersil ODS2 column (4.6 × 250 mm, 5 μm) and detection at 210 nm (HPLC-UV) [22].

Cisplatin was obtained from Sigma-Aldrich (St. Louis, MO, USA). Reagent kits: blood urea nitrogen (BUN), creatinine (CRE), and hematoxylin and eosin (H&E) staining were provided by Nanjing Jiancheng Bioengineering Research Institute (Nanjing, China). Terminal deoxynucleotidyl transferase dUTP nick end labeling (TUNEL) apoptosis detection kits were provided with Roche Applied Science in Shanghai, China (No. 11684817910). The primary antibodies of rabbit monoclonal anti-mouse such as iNOS, COX-2, TNF-α, IL-1β, b-associated X (Bax), b-cell-lymphoma-2 (Bcl-2), caspase-3, caspase-9, phosphatidylinositol 3-kinase (PI3K), p-PI3K, Protein kinase B (Akt), p-Akt, inhibitor of κBα (IκBα), p-IκBα, nuclear factor-kappa B (NF-κB), p-NF-κB, IκB kinase α/β (IKKα/β), p-IKKα/β, and β-actin were obtained from Cell Signaling Technology (Danvers, MA, USA). DyLight488-labeled and secondary antibodies were purchased from BOSTER Biological Technology (Wuhan, China). The remaining chemicals used in the experiments were analytical grade and bought from Beijing Chemical Factory.

### 2.2. Experimental Design

Male Institute of Cancer Research (ICR) mice, weighing between 20~22 g (eight weeks old) were purchased from Changchun YISI Experimental Animal Technology Limited Liability Company with Certificate of Quality No. of SCXK (JI) 2016-0003 (Changchun, China). The animals were maintained in a 12 h light/dark pattern with food and water ad libitum, at a constant temperature (23.0 ± 2.0 °C) and humidity (60 ± 10%). Before the experiment started, the mice underwent at least one week of acclimation period. The mouse experiments were approved by the Ethical Committee for Laboratory Animals of Jilin Agricultural University. The previous study by Jie Zheng et al. has proved the antihyperglycemic effects of *Platycodon grandiflorum* (Jacq.) A. DC. extract on streptozotocin-induced diabetic mice in a dose-dependent manner (150 and 300 mg/kg), and no side effects were observed in mice at a dose of 300 mg/kg [23]. Hence, we believe that PGS is safe for mice at doses of 15 and 30 mg/kg without side effects.

All mice were randomly divided into four groups with eight animals per group as follows:

1. Control group: Control mice were orally administered physiological saline daily for 10 days, with no drug treatment.
2. Cisplatin group: Mice were orally administered physiological saline daily for 10 days and received (25 mg/kg, i.p.) one hour after oral gavage on the seventh day.
3. PGS (15 mg/kg) + cisplatin group: Mice were orally administered 15 mg/kg PGS, which was dissolved in physiological saline, daily for 10 days and they received cisplatin (25 mg/kg, i.p.) one hour after PGS administration on the seventh day.
4. PGS (30 mg/kg) + cisplatin group: Mice were orally administered 30 mg/kg PGS, which was dissolved in physiological saline, daily for 10 days and they received cisplatin (25 mg/kg, i.p.) one hour after PGS administration on the seventh day.

Mice were anaesthetized with pentobarbital, and subsequently sacrificed at 72 h after cisplatin injection (Day 10). A blood sample was collected from eyeball. Serum was separated with centrifuged (1000× *g*, 10 min, 4 °C) and collected for biochemical analysis. The kidney tissues were then collected, washed with saline twice, and dried with filter paper before weighing. The left kidney was stored at −80 °C for later biochemical detection and Western blotting analysis. The right kidney tissue was fixed in 10% buffered formalin (V/V) for histological analysis, immunohistochemical staining, and immunofluorescence analysis.

### 2.3. Renal Function Tests

Serum markers of renal function, and serum BUN and CRE were determined by BUN and CRE assay kits according to the manufacturer’s instructions (Nanjing Jiancheng Institute of Biotechnology, Nanjing, China) [24]. The concentration of CRE in the serum was measured by the sarcosine oxidase method. A total of 20 μL of double distilled water was added to the blank wells, 20 μL of 10 mmol/L CRE standard application solution was added to the standard wells, and 20 μL of the sample to be tested was added to the wells. Then, 250 μL of buffer enzyme solution was added to each well, and the mixture was mixed and then incubated in a water bath at 37 °C for 10 min. A total of 1 mL of phenol developer and 1 mL of basic sodium hypochlorite were sequentially added to each well, and the mixture was thoroughly mixed, and then incubated in a water bath at 37 °C for 10 min, and measured at a wavelength of 546 nm. The concentration of BUN in the serum was measured by the urease method. A total of 6 μL of double distilled water was added to the blank wells, 6 μL of standard to the standard wells, and 20 μL of the sample to be tested to the wells. Then, 180 μL of enzyme solution A was added to each well, and after mixing, it was incubated at 37 °C for 5 min, and the absorbance value A1 was measured at a wavelength of 546 nm. Then, 60 μL of enzyme solution B was added to each well, mixed, and incubated for 5 min at 37 °C, and the the absorbance value A2 at 546 nm was measured.

### 2.4. Histopathology Analysis

H&E staining was performed with deparaffinization and dehydration of 5 μm-thick sections of renal tissues. Histopathology changes were observed by light microscope (Leica, Wetzlar, Germany). The necrotic degree was accessed by the necrotic area, as well as the inflammatory cell infiltration degree and congestion relative to the entire histological sections. Tubular injure scores were semi-quantitatively analyzed by counting the percent of tubules that displayed cell necrosis, tubule dilatation, loss of brush border, and cast formation, as follows: 0, none; 1, <10%; 2, 10% to 25%; 3, 25% to 75%; 4, >75%.

### 2.5. Immunohistochemical Staining

Immunohistochemical staining was performed as previously described [25]. Briefly, renal samples were routine dewaxed and antigen rehydrated, and then heating in citrate buffer (pH 6.0, 0.01 M), followed by rinsed with phosphate buffered saline (PBS) (0.01 M, pH 7.4) three times, and incubated with 1% bovine serum albumin (BSA) for 1 h. The sections were then incubated with primary antibodies, including mouse polyclonal anti-Bax (1:400) and -Bcl-2 (1:400) -iNOS (1:400) -COX-2 (1:400) overnight at 4 °C, followed by a secondary antibody for 30 min at room temperature. The slides were then treated with a dispute adjudication board (DAB) and followed by counterstaining with hematoxylin. A brown color was observed in the cytoplasm or the nucleus by light microscopy (Leica DM750, Solms, Germany).

### 2.6. Immunofluorescence Staining

The procedure of immunofluorescence staining is consistent with immunohistochemical staining. After the slices were incubated with primary antibody TNF-α (1:100) 4 °C for 12 h, the sections were exposed to Dylight448-labeled secondary antibody. Nuclei were visualized by 4,6 diamidino-2-phenylindole (DAPI) staining for 4 min, followed by three washes with PBS. The immunofluorescence intensity was observed by light microscope (Olympus BX-60, Tokyo, Japan).

### 2.7. Western Blotting Analysis

Protein samples were separated on preconfigured 12% sodium dodecyl sulphate - polyacrylamide gel electrophoresis (SDS-PAGE) and subsequently transferred to nitrocellulose membranes in prechilled electrophoretic transfer buffer. After transferred to polyvinylidene fluoride (PVDF) membrane, the target strip was rinsed 3 × 10 min with Tris-buffered saline with 0.1% Tween-20 (TBS-T). Next, membranes were blocked with 5% skim milk at room temperature for 2 h, followed by incubation with primary antibodies overnight at 4 °C. Subsequently, the membranes were washed in TBST and then incubated with pre-diluted secondary antibody for 1.5 h. The blots were detected by Emitter Coupled Logic (ECL) substrate (Pierce Chemical Co., Rockford, IL, USA). Protein band intensities were assessed with Quantity One software (Bio-Rad Laboratories, Hercules, CA, USA).

### 2.8. TUNEL Staining Analysis

To measure the extent of apoptosis in kidney after cisplatin exposure, the TUNEL analysis was determined. TUNEL evaluation was carried out as mentioned earlier with minor modification [26]. Typically, this was done by employing an in situ apoptosis detection kit (Mannheim, Germany) to discover apoptotic cells in the kidney tissues, according to the manufacturer’s instructions. Firstly, the kidney sections (5 μm thick) were installed on the slides and permeabilized by incubating them with 100 μL of 20 μg/mL proteinase K solution for 15 min. Next, the sections were incubated with 100 μL of 0.3% H_2_O_2_ for 5 min and incubated by equilibration buffer and terminal deoxynucleotidyl transferase to inactivate endogenous peroxidases. Then, anti-digoxigenin-peroxidase conjugates was employed to incubate the sections. Finally, the utilization of diaminobenzidine demonstrated peroxidase activity in all tissue sections, and the slices were counterstained with hematoxylin. TUNEL-positive cells were visualized with a Leica microscope (Leica TCS SP8, Solms, Germany).

### 2.9. Statistical Analysis

All data were presented as mean ± S.D. The statistical significance of mean values was assessed using a Student t-test and a one-way analysis of variance (ANOVA) followed by a Bonferroni post-hoc test. Statistics were performed using GraphPad Prism 6.0.4 software. A probability value of less than 0.05 or 0.01 was defined as statistically significant.

## 3. Results

### 3.1. PGS-Attenuated Cisplatin-Induced Renal Dysfunction and Renal Histopathological Changes in Mice

**Figure 1:**
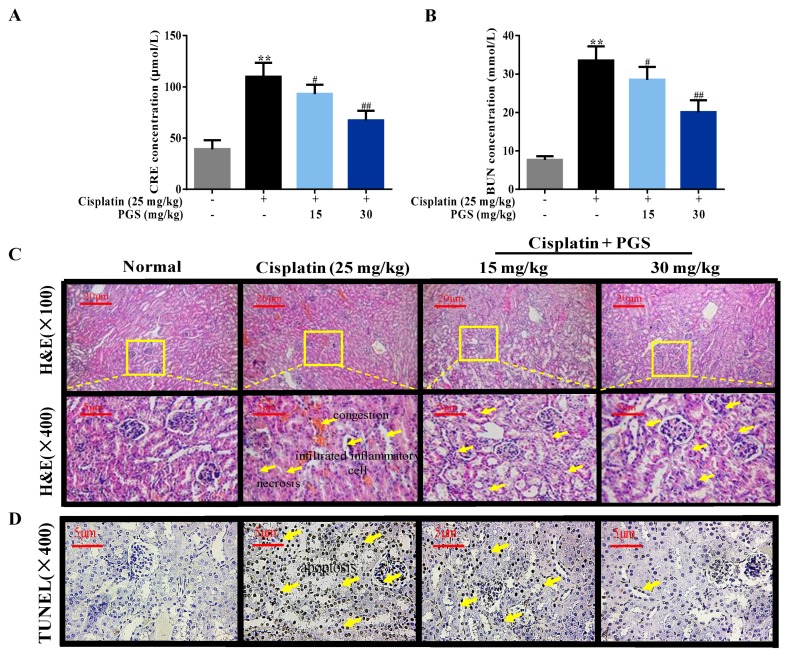

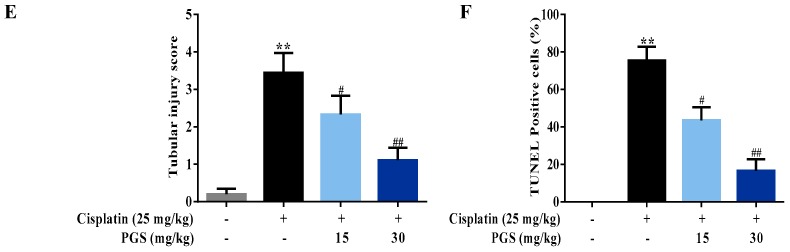
Renal protective effects of PGS against cisplatin-induced renal injury. Cisplatin increased serum CRE (**A**) and BUN (**B**) levels, whereas PGS reduced the increase. Kidneys stained with H&E (**C**) and TUNEL (400×) (**D**). The tubular injury scores (**E**) and the number of TUNEL-positive cells (**F**). ** *p* < 0.01 vs. Control group; ^#^ *p* < 0.05, ^##^ *p* < 0.01 vs. cisplatin group. CRE: creatinine, BUN: blood urea nitrogen, PGS: *Platycodon grandiflorum* saponins, H&E: hematoxylin-eosin staining, TUNEL: terminal deoxynucleotidyl transferase dUTP nick end labeling.

Serum CRE and BUN levels are closely linked to renal function. Compared to the control group, injection of cisplatin with 25 mg/kg highly elevated the serum CRE and BUN levels by 2.8- and 4.4- fold, respectively (*p* < 0.01), indicating the generation of nephrotoxicity in the cisplatin-treated mice. In contrast, pretreatment with PGS at doses of 15 and 30 mg/kg exerted a significant renoprotective effect, as demonstrated by the normalization of CRE and BUN (*p* < 0.05, *p* < 0.01) compared with the cisplatin group (Figure 1A,B).

In addition, we performed histopathological changes to explore whether PGS affects renal dysfunction in the presence of cisplatin. The renal tissues in the control group showed completely normal renal tissues, characterized by clear tubular and glomerular structures with clear and normal nuclei. Kidneys in the cisplatin-treated mice showed serious renal injury, manifested by tubular necrosis, tubular dilatation, and glomerular congestion. However, pretreatment with PGS markedly ameliorated necrotic and inflammatory infiltrated cells in the kidney tissues, especially at the higher dose of 30 mg/kg (*p* < 0.05, *p* < 0.01) (Figure 1C,E).

### 3.2. PGS Alleviated Cisplatin-Induced Apoptosis

**Figure 2:**
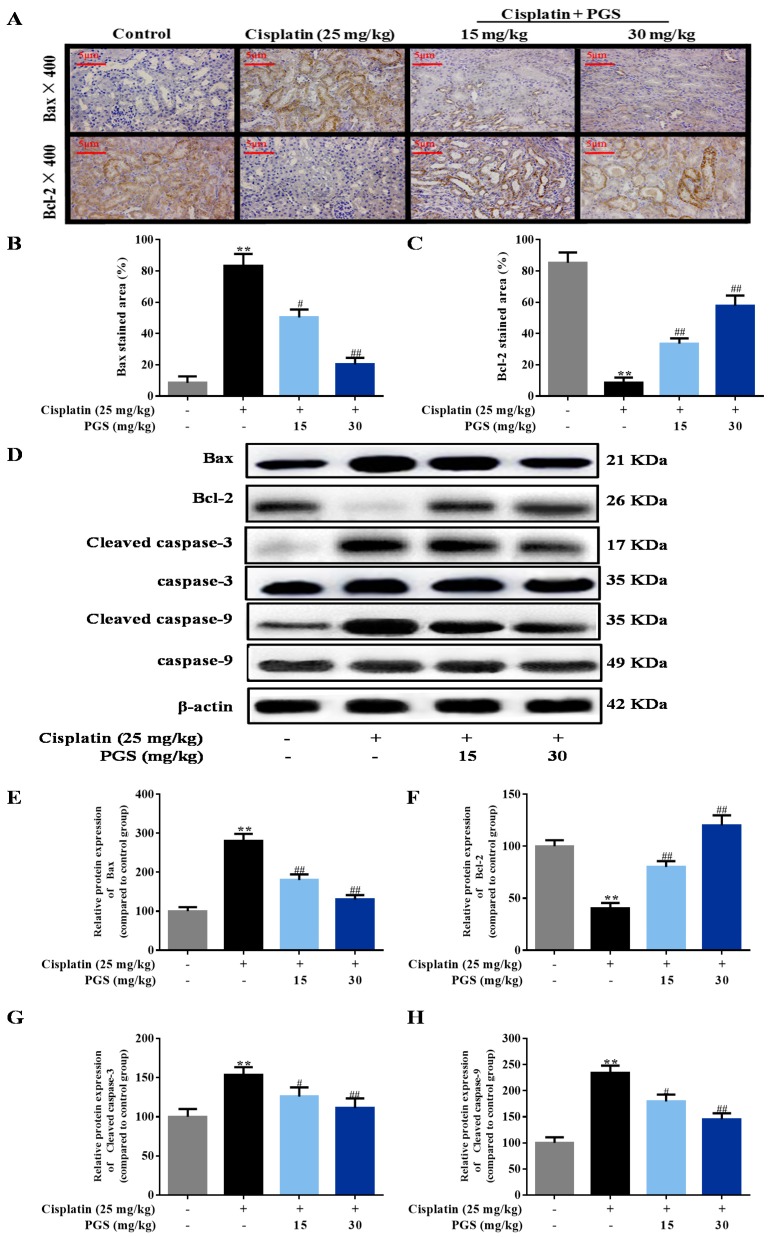
Effects of PGS pretreatment on apoptosis signaling pathways in cisplatin-triggered renal damage in mice. The protein expression of Bax and Bcl-2 (**A**) was examined by immunohistochemistry (**B**,**C**) in renal tissues, and the fluorescence intensities were quantified at 400×. 4,6 diamidino-2-phenylindole (DAPI) was used as a nuclear counterstain. The expression level of Bax, Bcl-2, cleaved caspase-3 and 9, and caspase-3 and 9 were measured by Western blotting (**D**). Quantitative analysis of scanning densitometry for Bax (**E**); Bcl-2 (**F**); cleaved caspase 3 (**G**); caspase 9 (**H**) were performed. ** *p* < 0.01 vs. control group; ^#^ *p* < 0.05, ^##^ *p* < 0.01 vs. cisplatin group. PGS: *Platycodon grandiflorum* saponins, Bax: b-associated X, Bcl-2: b-cell-lymphoma-2.

For exploring the mechanism of cisplatin-induced cell apoptosis in renal tissues, immunohistochemical staining was used to detect the apoptosis-related proteins Bax and Blc-2 in kidney sections. Exposure of cisplatin increased the expression of Bax by 9.9-fold, and decreased the expression of Bcl-2 by 9.9-fold in the kidney, respectively, when compared to the control group. In contrast, 10-day pretreatment with PGS (15, 30 mg/kg) greatly inhibited these changes. The above data indicated that the pre-administration with PGS was able to inhibit cell apoptosis triggered by cisplatin injection (*p* < 0.05, *p* < 0.01) (Figure 2A–C).

Expression of apoptosis-related markers such as Bax, cleaved caspase-3 and 9, and Bcl-2 in the renal samples were detected through Western blotting analysis. Cisplatin exposure increased the levels of Bax, and cleaved caspase-3 and 9 by 2.8-, 1.5-, and 2.3-fold in the kidney, respectively, when compared to the control group. Exposure of cisplatin decreased the expression of Bcl-2 by 2.5-fold in the kidney when compared to the control group, while the changes were clearly ameliorated by PGS pretreatment (*p* < 0.05, *p* < 0.01) (Figure 2D–H). These results indicated that PGS pre-treatment prevented apoptosis induced by cisplatin in renal tissues.

Renal cell apoptosis was determined and quantified through TUNEL staining, suggesting that apoptosis is accordant with necrosis after cisplatin exposure. As indicated in Figure 1D, almost no positive cells were visualized in the control mice. Interestingly, mice treated with cisplatin showed a higher amount of TUNEL-positive cells, compared to the control group, whereas PGS pretreatment (15 and 30 mg/kg) reversed this evaluation (*p* < 0.05, *p* < 0.01).

### 3.3. PGS Attenuated Cisplatin-Induced Renal Inflammation

**Figure 3:**
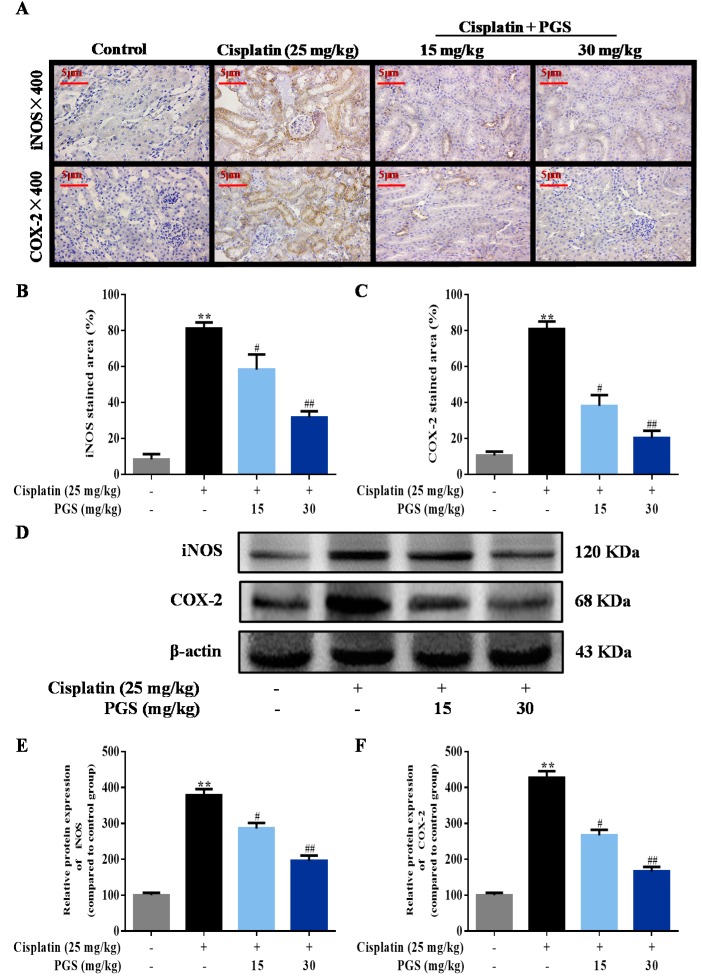
Effects of PGS pretreatment on cisplatin-induced inflammatory responses in mice with renal injuries. The expression of iNOS and COX-2 (**A**) was examined by immunohistochemistry (**B**,**C**) in renal tissues, and fluorescence intensities were quantified at 400×. 4,6 diamidino-2-phenylindole (DAPI) was used as a nuclear counterstain. The expression of iNOS and COX-2 were measured by Western blotting (**D**). Quantitative analysis of scanning densitometry for iNOS (**E**); COX-2 (**F**). ** *p* < 0.01 vs. control group; ^#^ *p* < 0.05, ^##^ *p* < 0.01 vs. cisplatin group. PGS: *Platycodon grandiflorum* saponins, iNOS: inducible nitric oxide synthase, COX-2: cyclooxygenase-2.

For analyzing whether PGS was able to protect renal cells from the cisplatin-induced inflammatory response, immunohistochemical staining methods was used to detect the inflammatory-related protein iNOS and COX-2. Exposure of cisplatin increased the levels of iNOS and COX-2 by 9.7- and 8-fold in the kidney, respectively, when compared to the control group. Mice treated with PGS exhibited the dose-dependent reduction in iNOS and COX-2 expressions. (*p* < 0.05, *p* < 0.01) (Figure 3A–C). In addition, reduction of the overproduction of iNOS and COX-2 in the renal tissues by the PGS pretreatment were confirmed by Western blotting analysis (Figure 3D–F) (*p* < 0.05, *p* < 0.01).

**Figure 4:**
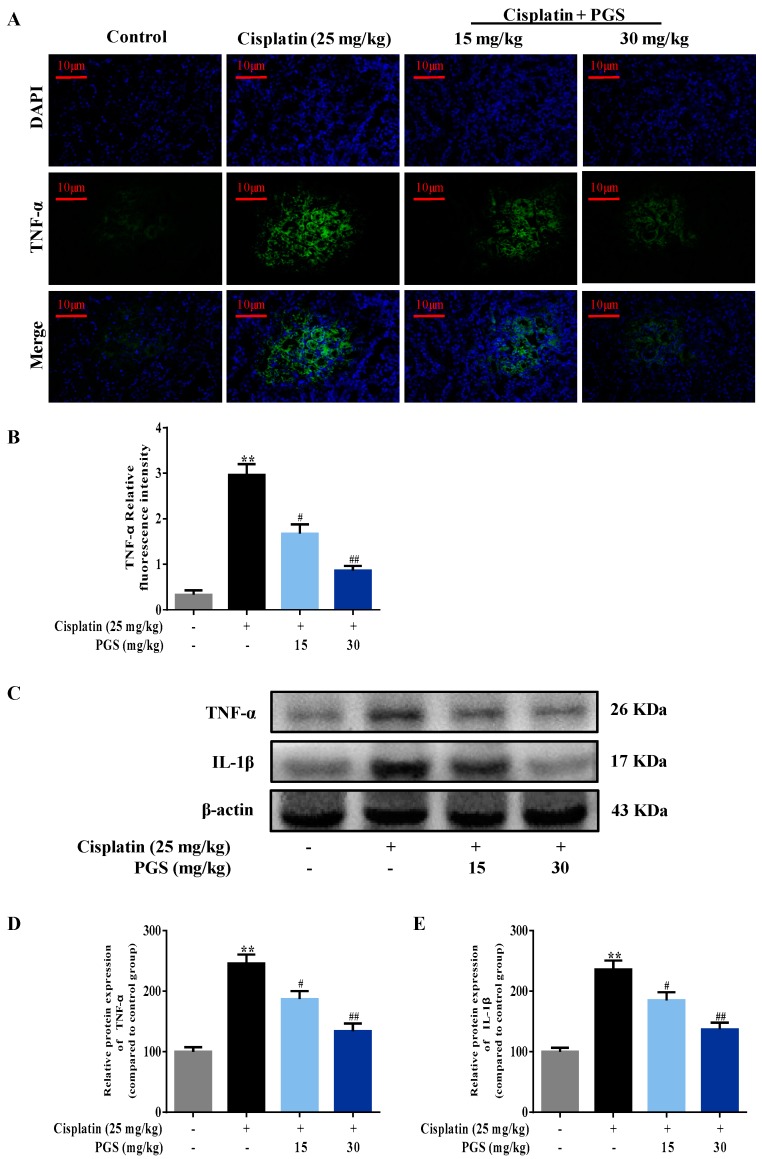
Effects of PGS pretreatment on cisplatin-induced inflammatory responses in mice with renal injuries. The expression of TNF-α (**A**) in renal samples of different groups was assayed by immunofluorescence. The fluorescence intensity of TNF-α (**B**) (green fluorescent) was quantified. Representative immunofluorescence images were taken at 400×. 4,6 diamidino-2-phenylindole (DAPI) was used as a nuclear counterstain. Protein expressions of TNF-α and IL-1β were measured by Western blotting (**C**). Quantitative analysis of scanning densitometry for TNF-α (**D**); IL-1β (**E**). ** *p* < 0.01 vs. control group; ^#^ *p* < 0.05, ^##^ *p* < 0.01 vs. cisplatin group. PGS: *Platycodon grandiflorum* saponins, TNF-α: tumor necrosis factor-α, IL-1β: interleukin-1β.

The expression of TNF-α in the renal tissues was measured by immunofluorescence intensity. Cisplatin exposure increased the expression of TNF-α by 8.9-fold in the kidney when compared to the control group. As indicated in Figure 4A,B, the expression levels of TNF-α induced by cisplatin was ameliorated by PGS (15, 30 mg/kg/day) pre-treatment (*p* < 0.05, *p* < 0.01). To further confirm our results, we detected the expressions of TNF-α and IL-1β in the renal samples by Western blotting analysis. Cisplatin exposure increased the expression of TNF-α and IL-1β by 2.47- and 2.34-fold in the kidney, respectively, when compared to the control group (Figures 4C–E) (*p* < 0.05, *p* < 0.01).

### 3.4. PGS Regulated the NF-κB Signaling Pathway

**Figure 5:**
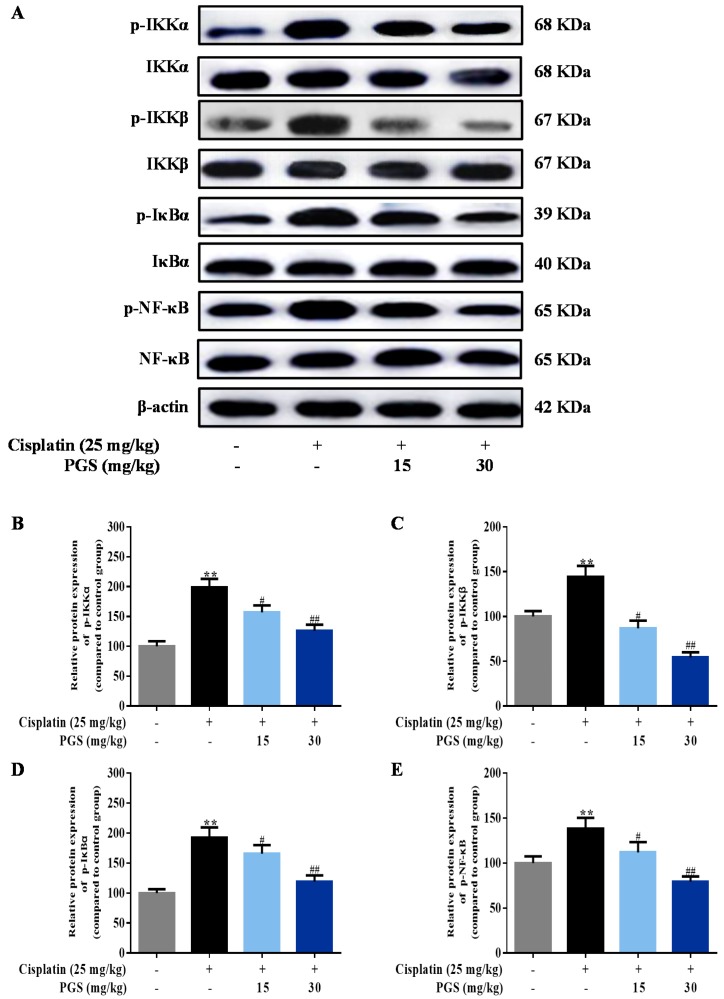
Effects of PGS treatment on the NF-κB signaling pathway against cisplatin-induced renal damage in mice. The expression levels of p-IKKα, IKKα, p-IKKβ, IKKβ, p-IκBα, IκBα, p-NF-κB, and NF-κB were measured by Western blotting (**A**). Quantitative analysis of scanning densitometry for p-IKKα (**B**); p-IKKβ (**C**); p-IκBα (**D**); p-NF-κB (**E**). ** *p* < 0.01 vs. control group; ^#^ *p* < 0.05, ^##^ *p* < 0.01 vs. cisplatin group. PGS: *Platycodon grandiflorum* saponins, IKKα: IκB kinase α, p-IKKα: phosphor-IKKα, IKKβ: IκB kinase β, p-IKKβ: phosphor-IKKβ, IκBα: inhibitor of κBα, p-IκBα: phosphor-IκBα, NF-κB: nuclear factor-kappa B, p-NF-κB: phosphor-NF-κB.

The NF-κB signaling pathway is a classic inflammatory pathway, and it promotes the release of inflammatory factors in the cisplatin-induced renal injury model of mice. The effects of PGS treatment on cisplatin-activated NF-κB signal pathway were tested by Western blotting analysis in this study. Levels of NF-κB and its upstream regulators, including IKKα/β and IκB, were increased by cisplatin treatment. Pretreatment with PGS (15, 30 mg/kg) significantly reduced the phosphorylation of IKKα/β and IκB and sequestered the phosphorylation of NF-κB, which effectively extenuated cisplatin-induced renal inflammation (*p* < 0.05, *p* < 0.01) (Figure 5A–E).

### 3.5. PGS Regulated the PI3K/Akt Signaling Pathway

**Figure 6:**
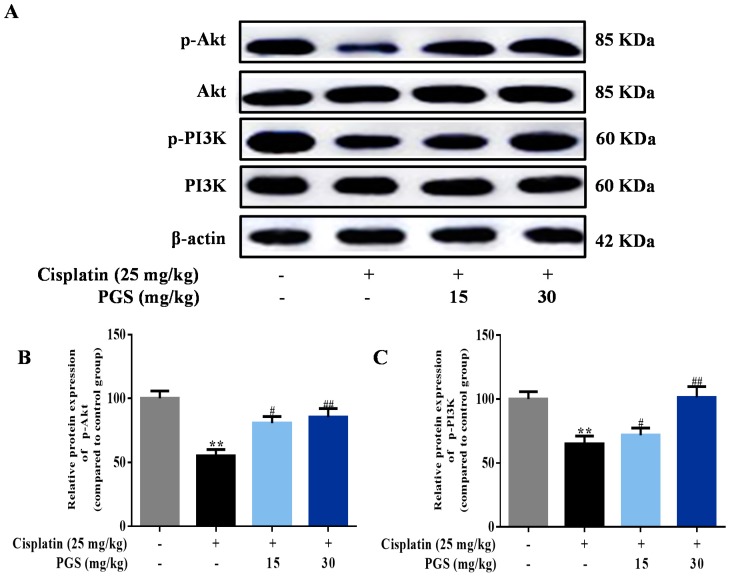
Effects of PGS pretreatment on the PI3K/Akt signaling pathway against cisplatin-caused renal damage in mice. The expression levels of p-PI3K, PI3K, p-Akt, and Akt were measured by Western blotting (**A**). Quantitative analysis of scanning densitometry for p-Akt (**B**); p-PI3K (**C**). ** *p* < 0.01 vs. control group; ^#^ *p* < 0.05, ^##^ *p* < 0.01 vs. cisplatin group. PGS: *Platycodon grandiflorum* saponins, Akt: Protein kinase B, p-Akt: phosphor-Akt, PI3K: phosphatidylinositol 3-kinase, p-PI3K: phosphor-PI3K.

The PI3K/Akt pathways exert protective effects against cisplatin-caused renal toxicity [27]. For exploring the molecular mechanism of PGS against cisplatin-induced renal toxicity, mice were co-treated with PGS and cisplatin for detect the expression of PI3K and Akt. Cisplatin-treatment remarkably reduced the expressions of phosphorylated PI3K and Akt. However, the phosphorylation levels of PI3K and Akt were increased by PGS pretreatment (*p* < 0.05, *p* < 0.01) (Figure 6A–C).

## 4. Discussion

Cisplatin is a commonly used chemotherapeutic agent against numerous solid tumors [28]. Unfortunately, cisplatin-induced nephrotoxicity is a significant limiting factor for its clinical utility [29]. Cisplatin-induced DNA fragmentation, apoptosis, and inflammation response are essential underling mechanisms for its toxic effects on renal tissues [30]. Therefore, compensation of cisplatin-induced toxicity on control tissues is urgently needed.

Currently, cisplatin-induced renal injury has been treated with natural chemical constituents extracted from Chinese herbs both in vivo [31] and in vitro [32]. PGS and triterpenoid saponins isolated from the roots of *P. grandiflorum* have the advantage of a simple extraction method with low costs. Studies have illustrated the anti-inflammatory and anti-apoptotic activities of PGS in multiple organs [33,34]. Although several studies have clearly clarified that Platycodin D (PD) as a maker of saponin was used to protect against cisplatin-induced nephrotoxicity in mice, there is currently no simple and rapid method to isolate PD. Hence, PGS as crude saponins is more economical and efficient, and is beneficial for industrial production [35]. Furthermore, the mechanism of PGS against cisplatin-induced renal injury remains poorly elucidated. Considering the protective effects of PGS against several tissue injuries, we hypothesize that PGS prevents cisplatin-induced nephrotoxicity via suppressing renal inflammation response and apoptosis.

The kidney is a critical organ that maintains a proper circulating ionic environment. It is the first organ to respond to disturbance in the circulating ionic milieu, and to adverse hits by chemicals and drugs in circulation. In this study, pretreatment with PGS for 10 consecutive days improved cisplatin-induced renal histopathological changes and decreased serum CRE and BUN levels (reflection of renal injury) in mice.

In the pathogenesis of cisplatin-induced nephrotoxicity, inflammation, besides direct cellular toxicity, has been recognized as a crucial contributor. Specifically, cisplatin promotes the transcription of inducible inflammatory factors and enzymes, which includes TNF-α, IL-1β, COX-2 and iNOS via activating the NF-κB pathway [36]. TNF-α further contributes to cisplatin-induced renal injury via activating the inflammation response, which increases the major damage [37]. IL-1β is also involved in inflammation, and it expresses excellent pro-inflammatory activities via increasing the expression of adhesion molecules and promoting stromal cells to release chemokines that stimulate the aggregation of inflammatory cells [37]. PGS was reported to down-regulate iNOS and COX-2 through the inhibition of NF-κB in high fat diet-fed rats [38]. In our current research, PGS diminished the TNF-α- and IL-1β-induced inflammation response, as determined by immunohistochemical staining, immunofluorescence staining, and Western blotting analysis. These changes were also accompanied by a decrease in iNOS and COX-2 levels, suggesting suppression of inducible enzymatic pathways. These current findings support a potential role for PGS in improving the inflammation response of cisplatin-induced renal injury.

NF-κB-mediated inflammation has been demonstrated to exert important effects in the pathogenesis of nephrotoxicity induced by cisplatin. The migration of subunit p65 and the activation of IκBα together activate the transcription of NF-κB [39]. IκBα here is rapidly phosphorylated and degraded after the classical activation of NF-κB signaling, which further leads to a release of NF-κB subunits [39]. After activation, NF-κB dissociates from IκBα and translocates from the cytoplasm to the nucleus where it initiates transcriptions of specific target genes, TNF-α, IL-1β, iNOS, and COX-2 [40]. In this study, PGS suppressed NF-κB activation in cisplatin nephrotoxicity, evidenced by decreased expressions of p-IKKα, p-IKKβ, p-IκBα, and p-NF-κB.

It is reported that PI3K participates in promoting endothelial progenitor cell proliferation and differentiation [41]. PI3K is an intracellular phosphatidylinositol kinase that regulates cell growth, proliferation, differentiation, motility, and survival [42]. Akt protein is known as protein kinase PKB, an important downstream target of the PI3K signaling pathway, and it takes good effect against cell apoptosis [43]. PI3K/Akt signaling pathways are vital in protecting cells against apoptosis [44]. The PI3K/Akt pathways were inhibited by cisplatin in kidney tubular epithelial cells [45]. Kuwana et al. has shown that the inhibition of PI3K induced more cell apoptosis when exposed to cisplatin treatment, showing that the increased expressions of PI3K/Akt pathway may be a target for renal protection [11].

Furthermore, the protein Bax- and Bcl-2-dependent mitochondrial apoptosis cascade is the main pathway in cisplatin-induced apoptosis [46]. Bcl-2 suppresses cytochrome c release from mitochondria triggered by the pro-apoptotic molecule Bax, leading to the block of caspase release and apoptosis [47]. The caspase family is a protease family that leads directly to the disintegration of apoptotic cells, and disturbs the balance of renal function [48]. Caspase-9 is one of the most important apoptotic promoters. It undergoes self-agitation in the presence of other proteins, and activates downstream caspase factors, such as caspase-3, which then cause a cascade-amplifying effect, leading to cell apoptosis [49]. Caspase-3 is not only one of the most important apoptotic executors in the caspase family, but it is also a major effector in the process of apoptosis, and its activation marks that apoptosis enters the irreversible phase [50]. In this study, cisplatin administration suppressed the expressions of p-PI3K and p-Akt, which lead to changes in Bax and Bcl-2 expression, and the activation of caspase-3 and caspase-9. In addition, the results of TUNEL staining show that the apoptosis rate in renal tissues were clearly reduced by PGS when compared with the cisplatin control group. Pretreatment of mice with PGS exerted an anti-apoptotic effect in a dose-dependent manner against cisplatin-induced renal apoptosis.

In summary, the PGS pretreatment of mice improves the cisplatin-induced renal injury by diminishing NF-κB-mediated inflammation and the PI3K/Akt/apoptosis signaling pathways. PGS are considered as renoprotective natural compounds against cisplatin-induced renal injury.
